# Progress and Challenges Toward Generation and Maintenance of Long-Lived Memory T Lymphocyte Responses During COVID-19

**DOI:** 10.3389/fimmu.2021.804808

**Published:** 2022-02-17

**Authors:** Swatantra Kumar, Shailendra K. Saxena, Vimal K. Maurya, Anil K. Tripathi

**Affiliations:** Centre for Advanced Research (CFAR), Faculty of Medicine, King George’s Medical University (KGMU), Lucknow, India

**Keywords:** SARS-CoV-2, COVID-19, T cell response, B cell response, immunopathogenesis, cytokine storm, memory T cell response

## Abstract

Severe acute respiratory syndrome coronavirus 2 (SARS-CoV-2) causing the coronavirus disease 2019 (COVID-19) pandemic is a serious global threat until we identify the effective preventive and therapeutic strategies. SARS-CoV-2 infection is characterized by various immunopathological consequences including lymphocyte activation and dysfunction, lymphopenia, cytokine storm, increased level of neutrophils, and depletion and exhaustion of lymphocytes. Considering the low level of antibody-mediated protection during coronavirus infection, understanding the role of T cell for long-term protection is decisive. Both CD4^+^ and CD8^+^ T cell response is imperative for cell-mediated immune response during COVID-19. However, the level of CD8^+^ T cell response reduced to almost half as compared to CD4^+^ after 6 months of infection. The long-term protection is mediated *via* generation of immunological memory response during COVID-19. The presence of memory CD4^+^ T cells in all the severely infected and recovered individuals shows that the memory response is predominated by CD4^+^ T cells. Prominently, the antigen-specific CD4^+^ and CD8^+^ T cells are specifically observed during day 0 to day 28 in COVID-19-vaccinated individuals. However, level of antigen-specific T memory cells in COVID-19-vaccinated individuals defines the long-term protection against forthcoming outbreaks of SARS-CoV-2.

## Introduction

The global emergence of Severe acute respiratory syndrome coronavirus 2 (SARS-CoV-2) caused the pandemic coronavirus disease (COVID-19), affecting at least 243 million cases with 4.9 million deaths (1). SARS-CoV-2 belongs to *Coronaviridae* which is a family of diverse enveloped RNA viruses of positive sense (2). SARS-CoV-2 is primarily transmitted *via* direct, indirect, or close contact with respiratory droplets generated by infected individuals through sneezing, coughing, talking, or singing. However, other possible routes of transmission occur *via* fomite, blood-borne, fecal–oral, mother-to-child, and animal-to-human transmission (3). Among the coronaviruses (CoVs), SARS-CoV-2 is the seventh coronavirus that infected humans. The size of the SARS-CoV-2 genome ranges from 27 to 32 kb which comprises of 6–11 open reading frames (ORFs). Among all the ORFs, the replicase (ORF1a/ORF1b), spike (S), membrane (M), envelope (E), and nucleocapsid (N) are the six functional ORFs, whereas seven putative ORFs encode for accessory proteins, which are interspersed in between the structural genes. The replicase gene encompasses 67% of the genome that encodes for a large polyprotein (pp1ab) that gets processed into 16 non-structural proteins (nsps) (4, 5). SARS-CoV-2 infection initiates upon attachment of spike glycoprotein with the ACE2 receptor and consequent priming of spike protein through host cell serine protease TMPRSS2 (6). Following entry, viral RNA is released into the cytoplasm which instantly undergoes translation to generate ORF1a and ORF1b (7). Crystal structures of crucial SARS-CoV-2 proteins have been resolved which are crucial for the designing of effective therapeutic strategies (8–11). However, understanding of SARS-CoV-2 immunopathology and immune response is crucial for designing effective vaccines and immunotherapeutics (12). Importantly, understating of effector T and B cell response is vital for controlling SARS-CoV-2 infection and crucial to providing long-lasting protection *via* generation of antigen-specific immunological memory response (13, 14). This strategy may help us to implement more effective vaccines in the mass population for reducing the burden of COVID-19 (15, 16).

## Immunopathology of SARS-CoV-2 Infection

COVID‐19 represents a complex profile with heterogeneous clinical manifestations (17). Most of the SARS-CoV-2 infections are asymptomatic and may present mild to moderate clinical symptoms of upper respiratory tract whereas around 15% of the cases result in severe pneumonia and approximately 5% of the cases result in acute respiratory distress syndrome (ARDS) or multiple-organ dysfunction due to septic shock (18). Severe patients are identified with bilateral lung involvement where 80% of the severe cases necessitate oxygenation, of which 30%–40% need mechanical ventilation. Importantly, 80%–90% of the severe cases of mechanical ventilation is the prime cause of COVID-19-associated mortality (19). Critically ill or severe COVID-19 patients are characterized as lymphocyte activation identified as increased levels of CD38, CD69, and CD44 T cell activation markers and exhaustion of T cells identified as the increased expression of T cell immunoglobulin domain and mucin domain-3 (TIM3), programmed cell death protein-1 (PD1), and killer cell lectin-like receptor subfamily C member 1 (NKG2A) (20). Therefore, lymphopenia or lymphocytopenia has been found as a decisive feature of severe cases of COVID-19 (21). In addition, the level of neutrophils is strikingly higher whereas the levels of monocytes, eosinophils, and basophiles have been found to be reduced (22). Importantly, severe cases of COVID-19 are identified as uncontrolled inflammatory response known as cytokine storm where IL-6, IL-1β, IL-10, and IFN-γ have been found to be significantly higher (23). Moreover, patients are identified with the higher level of immunoglobulin G (IgG) and total antibodies (24). Non-survivors are identified as elevated levels of C-reactive proteins, serum ferritin, lactate dehydrogenase, and serum IL-6 as compared with survivors (25). Analysis of the postmortem samples showed the infiltration of lymphocytes and macrophages in the lungs as well as hemophagocytosis in reticuloendothelial organs and bone marrow (26). The SARS-CoV-2-induced lung injury is characterized by diffuse alveolar damages in the pulmonary vessels identified as platelet fibrin microthrombi (27, 28). Altogether, this hyperinflammatory response during COVID-19 suggests the involvement of diverse COVID-19 immunopathologies and host immune responses. Classification of the severity of the disease is crucial for the gradient-based treatment of COVID-19. So far, the radiological imaging of pulmonary systems and other auxiliary examinations are exhibited for the classification of the disease severity (29). However, the blood profiling of the patients is a cost-effective examination of the severe cases.

## Cytokine Storm During COVID-19

**Figure 1:**
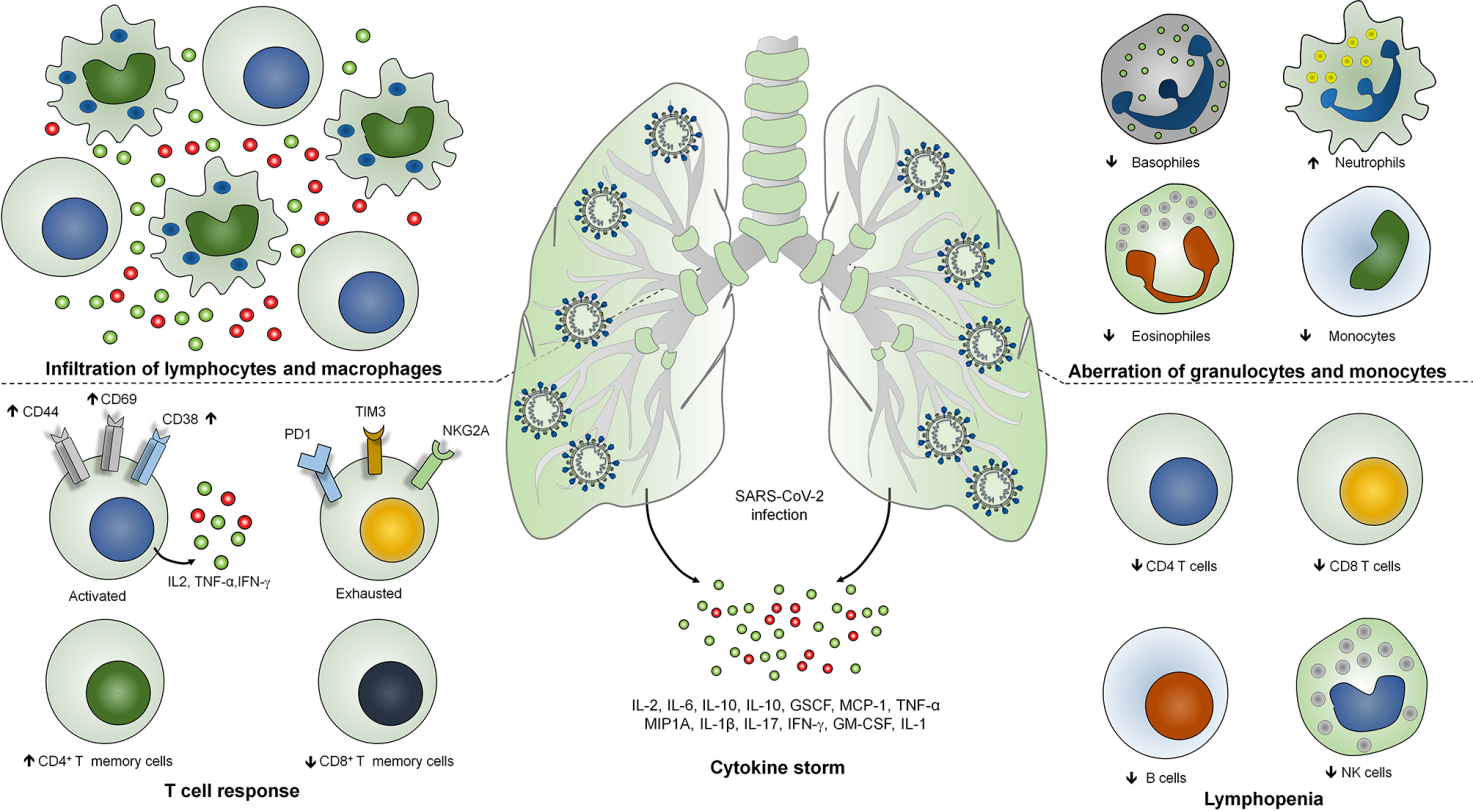
Immunopathogenesis of SARS-COV-2 infection. Severe COVID-19 is identified with infiltration of lymphocytes and macrophages in the lungs, lymphopenia or lymphocytopenia where reduction in the number of CD4^+^, CD8^+^ T cells, B cells and NK cells occurs. SARS-CoV-2 infection is identified with the lymphocyte activation with increased expression of CD38, CD69 and CD44 activation markers and T cell exhaustion identified as T cell immunoglobulin domain and mucin domain-3 (TIM3), programmed cell death protein-1 (PD1), and killer cell lectin-like receptor subfamily C member 1 (NKG2A) markers. Level of neutrophils is strikingly higher whereas the level of monocytes, eosinophils and basophiles has been found to be reduced. Severe COVID-19 patients have been shown to exhibits higher levels of IL-2, IL-6, IL-10, IL-1, GSCF, MCP-1, TNF-α, MIP1A, IFN-γ, IL-1 and GM-CSF.

In addition to T and B cell response, elevated levels of cytokines are associated with the disease severity and mortality during SARS-CoV-2 infection (30). Activation of coagulation pathways as an immune response mechanism against SARS-CoV-2 infection is associated with the hyperactivation of proinflammatory cytokine production and multi organ failures (31). Importantly, some of the crucial cytokines such as CCL7, CXCL10, and IL-1 receptor antagonist are associated with the increased viral load, pulmonary dysfunction and damage, and mortality (32, 33). Severe COVID-19 patients have been shown to exhibit higher levels of IL-2, IL-6, IL-10, IL-1, GSCF, MCP-1, TNF-α, and MIP1A (34, 35). Interestingly, the peak plasma levels of IL-6 have been shown to be less as compared with the patients with hyperinflammatory ARDS, cytokine release syndrome, and sepsis (**Figure 1**) (36).

## T Cell Response During COVID-19

Similar to other respiratory viral infections, lymphocyte response is crucial during SARS-CoV-2 infection. Response of lymphocytes specifically T cells is vital since the cellular immune response is exhibited *via* T cells which are involved in the direct killing of the virus-infected cells *via* cytotoxic T lymphocyte CD8^+^ T cells and CD4^+^ T cell-mediated CD8^+^ T cell priming and induction of B cell differentiation into plasma cells to produce virus-specific antibodies (37). Response of both T and B cells during SARS-CoV-2 infection has been detected in the blood approximately 1 week after the onset of symptoms (38). Studies are suggesting that the activation of CD8^+^ T cells is greater than the CD4^+^ T cell response which was observed by the higher expression of activation markers HLA-DR and CD38 (39). Response of a high magnitude of CD8^+^ T cells was observed in the mild cases of COVID-19 which may define the protective role of these cells (40). However, severe cases of COVID-19 have been shown to exhibit the terminally differentiated or exhausted CD8^+^ T cells with an increased expression of inhibitory receptors including TIM3, PD1, CTLA4, LAG3, CD39, and NKG2A showing the characteristic of T cell dysfunction. However, some reports are suggesting that SARS-CoV-2-infected individuals exhibit functional CD8^+^ T cells identified by PD-1 (41). These data also suggest the hyperactivation of antigen-specific CD8^+^ T cells might be the cause of disease severity (42). Interestingly, CD4^+^ T cell response has been observed against spike glycoprotein in the recovered patients (43) whereas CD8^+^ T cell response was specifically attributed toward the internal proteins of SARS-CoV-2 (44). The six predominant epitopes have been identified to be involved in the T cell response where three epitopes are from spike and two from membrane proteins and one is from nucleocapsid (45). In addition, a significant CD4^+^ T cell response was found to be specific against spike, nsp3, nsp4, ORF3s, ORF7a, nsp12, and ORF8 (46).

## B Cell Response During COVID-19

In patients of COVID-19, B cell response has been found to be elicited against nucleocapsid protein which concomitantly exhibited with T follicular helper cell response after 1 week of onset of symptoms (47). Strikingly, antibody response against spike glycoprotein was observed 4–8 days after the onset of symptoms (38, 48). Neutralizing antibody response against spike glycoprotein was found to be generated after 2 to 3 weeks (49). A subset of individuals has been found to be incapable of developing long-lasting antibody response and therefore might be prone to the reinfections (50, 51). Importantly, SARS-CoV-2 infection has been shown to be involved in antibody-dependent enhancement of infection mediated by IgG receptors FcγRIIA and FcγRIIIA (52). Strikingly, despite of the presence of the anti-SARS-CoV-2 RBD antibody of serum neutralization activity, effector B cell response is linked with poor clinical outcome and disease severity of the COVID-19 patients (53). The B cell response during SARS-CoV-2 infection mimic the patients reported with active autoimmune processes and human systemic lupus erythematosus (54). In spite of productive humoral response marked by higher antibody-secreting cells (ASCs), expansion was associated with the more severe infections in a subset of COVID-19 patients; neutralizing antibodies were found to provide ineffective protection against SARS-CoV-2 infection (55).

## Lymphopenia in Severe COVID-19

Lymphopenia or lymphocytopenia has been found as the key immunopathological characteristic of severe COVID-19 cases where 20% of the severe cases showed a low T cell count (56). More specifically, CD8^+^ T cells remains low as a result of COVID-19-associated lymphopenia (57). In addition, the level of memory T_H_ cells identified as triple-positive cells (CD3^+^CD4^+^ and CD45RO^+^) has been found to be reduced (58). The probable reason for the lymphopenia has been suggested *via* four ways, namely, SARS-CoV-2 directly infecting the lymphocytes causing lymphocyte programmed cell death, damage to the lymphoid organs, hyperinflammation-mediated lymphocyte dysfunction probably *via* TNF-α and IL-6, and metabolic molecule-mediated lymphocyte dysfunction (59). All of the probable mechanisms result in lymphopenia, and therefore, admitted patients may be immediately subjected to T cell count which suggests the severity of the case (60).

## Memory T Cell Response During SARS-CoV-2 Infection

Although neutralizing antibody response is important for protection against SARS-CoV-2 infection, long-term protection is required from the onset of infection during reexposure of infection and therefore is crucial for designing effective vaccine candidates for COVID-19 which aims to generate robust memory response upon reexposure. Memory T and B cell responses are the most vital immunological responses, which provide long-term protection against any infections. Recovered COVID-19 patients have been shown to exhibit robust and broad memory CD4^+^ and CD8^+^ T cell responses (45, 61). Human peripheral CD4^+^ T cells can be classified based on their activity during antigen reexposure where naïve cells can be characterized as CCR7^+^ and CD45RA^+^ cells, and central and effector memory cells are characterized as CCR7^+^ CD45RA^-^ and CCR7^-^ CD45RA^-^, respectively. Recovered convalescent patients who were recently discharged from the hospital and 2 to 4 weeks after being declared virus-free have been shown to exhibit persistent memory CD4^+^ T cell response as well as effector memory-circulating T follicular helper (cTfh) cells (47, 62). Unlike SARS-CoV infection, where memory CD8^+^ T cell response was found to be higher as compared with the memory CD4^+^ T cell response which persists for more than 6 years (63, 64), SARS-CoV-2-infected recovered patients showed memory CD4^+^ T cells in all patients where memory CD8^+^ T cells are present in 70% individuals, suggesting that memory response in severe cases is predominated by CD4^+^ Tm cells (45). In addition, all memory T cell responses have been observed against structural proteins of SARS-CoV after 9 and 11 years of recovery (65).

## Generation of Antigen-Primed T Cells During Natural COVID-19 Infection

Antigen-primed T cells are crucial to effectively countering the SARS-CoV-2 infection mediated by both CD4^+^ and CD8^+^ Teff cells (66). T cell response investigated against nucleocapsid (N) protein has been shown to exhibit robust IFN-γ response after 17 years of SARS-CoV infection against N peptides. Interestingly, PBMCs collected from these individuals elicited a similar response against N peptides from SARS-CoV-2. SARS-CoV-2-infected individuals exhibit N protein-specific T cell repertoires which are the part of individuals with a history of SARS-CoV infection (67). Prominently, T cell response has been investigated in COVID-19 patients 6 months after the infection. IFN-γ ELISPOT analysis revealed the presence of predominant antigen-specific CD4^+^ T cells with robust IL-2 expression (68). However, CD8^+^ response was found to be half as compared to the CD4^+^ T cell response which is mostly seen against non-spike proteins (68, 69). The level of T cell response can be strongly correlated with the magnitude of peak antibody level specific against spike and RBD (68). These data suggest that infection with betacoronaviruses induces long-lasting T cell-mediated immunity that will prevent COVID-19 survivors to be infected from forthcoming severe infections.

## Lymphocyte Response Against COVID-19-Vaccinated Individuals

**Figure 2:**
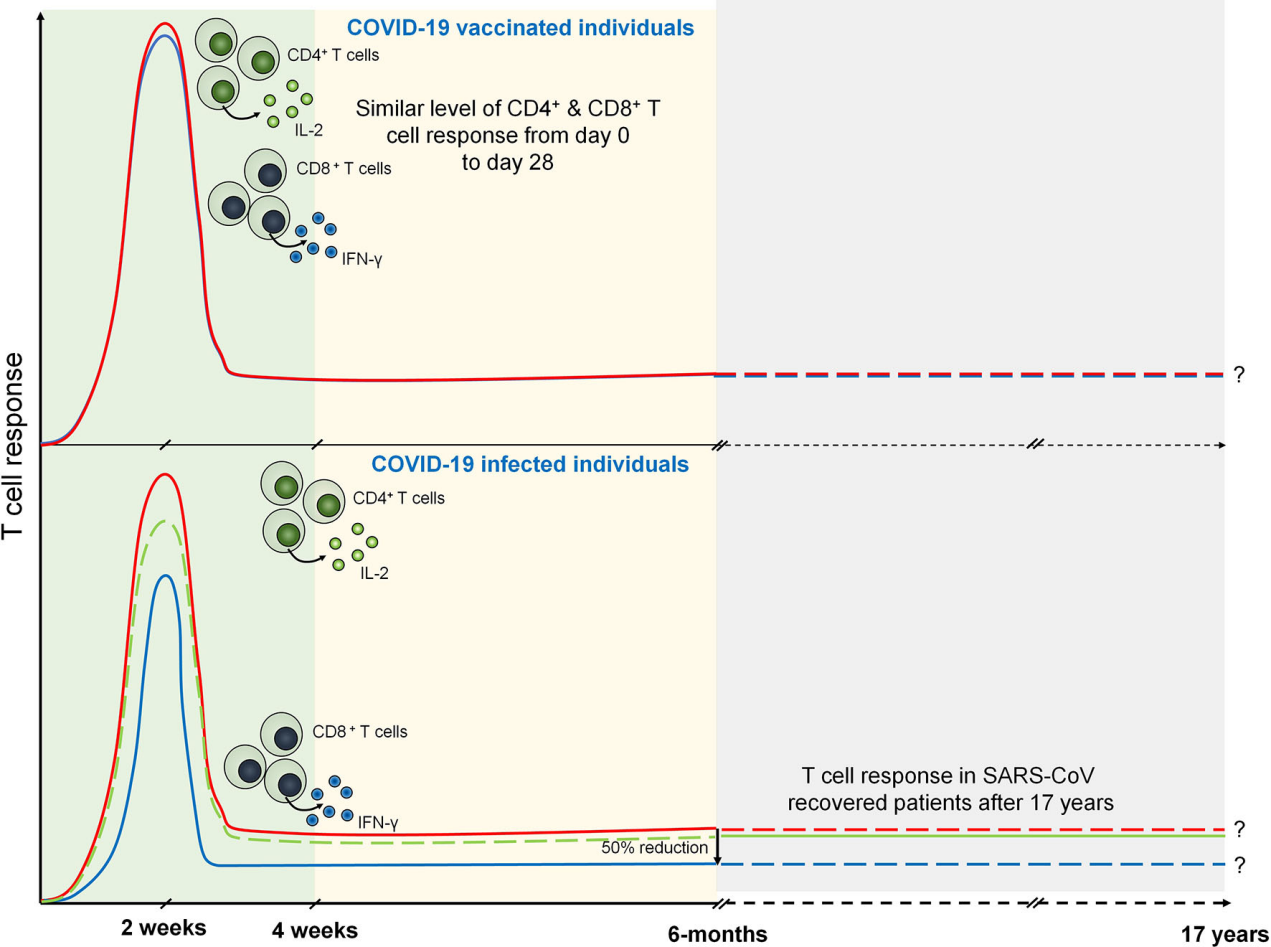
T cell response during COVID-19 and vaccinated individuals. Both CD4^+^ (marked by IL-2 expression) and CD8^+^ T (marked by IFN-γ expression) cell response are peaked at 14 days from the onset of infection. However, after 6 months of COVID-19 infection CD8^+^ T cell response becomes half of the CD4^+^ T cell response. SARS-CoV specific T cell response is seen after 17 years that may suggests the persistence of antigen specific memory T cells. Similarly, T cell response are seen in COVID-19 vaccinated individuals which are prominently observed in day 0 to day 28.

Considering the crucial role of T cell-mediated immunity during SARS-CoV-2 infection, it is imperative to understand the T cell response during COVID-19 vaccination. A replication-deficient simian adenoviral vector-based vaccine, ChAdOx1 nCoV-19 (AZD1222), has been shown to induce discrete clusters of populations of lymphocyte (70). These clusters were identified as Ki-67^+^ as proliferating population and CD69^+^ as activated population for both CD4^+^ and CD8^+^ T cells. Interestingly, terminally differentiating T cells identified as CD57^+^ and KLRG1^+^ were not detected showing a reduced low cytotoxicity response upon AZD1222 vaccination. Upon vaccination, anti-SARS-CoV-2 spike IgG1 and IgG3 responses have been detected at day 14 that further increased by day 28. However, by day 56, these responses were found to be similar to those by day 14. Importantly, IgG1 responses were detected in half, whereas IgG3 was detected in almost all the recruited vaccinated individuals (71). Similar to the COVID-19 patients, T cell responses were measured in AZD1222-vaccinated individuals using IFN-γ ELISpot assay which was peaked at day 14. Spike-specific cytokine response measured by intracellular cytokine staining (ICS) showed that CD4^+^ T cell response is heavily responsive toward secretion of Th1 cytokines specifically IL-2 and IFN-γ (**Figure 2**). Assessment of combination of cytokines shows that these responses are primarily dominated by monofunctional IFN-γ CD8^+^ T cells (71).

Considering the emergence of newer SARS-CoV-2 variants including Omicron, the efficacy of COVID-19 vaccines is crucial to be explored (72). Prominently, sera collected from the second dose of BNT162b2-vaccinated individuals show neutralizing Abs response against several of the emerging SARS-CoV-2 variants (73). Importantly, detection of IFN-γ, IL-12p70, and IL-2 but not IL-4 or IL-5 showed the promising T_H_1 response along with the absence of deleterious T_H_2 immune response (74). Likewise, the mRNA-1273 vaccine has been shown to elicit strong CD4 cytokine response specifically type 1 helper T cells (75). Importantly, no correlation of T cell response has been found in these vaccinated individuals toward common cold coronaviruses (CCCs). These vaccines show enhanced T cell response toward peptides derived from SARS-CoV-2 spike as well as toward HCoV-NL63 spike peptides (76).

## Conclusions

Severe COVID-19 cases are associated with the macrophage and lymphocyte infiltration to the lungs, which results in lung injury *via* activation of lymphocytes and hyperinflammatory responses. The T cell response is crucial as compared with the B cell response due to inability of antibody-mediated neutralization of SARS-CoV-2 and its level of reduction in long-term protection. Memory CD4^+^ T cell response is higher as compared with the memory CD8^+^ T cell response which might be linked with the compromised long-lasting protection. Considering the presence of memory T cell responses against structural proteins of SARS-CoV after 9 and 11 years of recovery, long-term protection against COVID-19 depends upon the presence of antigen-specific memory T cell response against SARS-CoV-2 which is predominated by CD4^+^ T cells. However, lymphopenia has been also reported in various severe infections, which results in compromised immune response and death. COVID-19-vaccinated individuals show presence of antigen-specific CD4^+^ and CD8^+^ T cells observed on day 0 to day 28. However, the level of these cells in COVID-19-vaccinated individuals defines the long-term protection against forthcoming outbreaks of SARS-CoV-2.

## Future Perspectives

The number of SARS-CoV-2 infection is continuously rising with the emergence of more mutant strains. A comprehensive understanding of immunological response is crucial for the development of effective immunotherapy and vaccines. Considering the inability of complete protection mediated by antibody response, understanding of memory cell response during SARS-CoV-2 infection is crucial for developing the effective vaccine for long-term protection. Therefore, more clinical studies are required that focus on the memory T cell response during COVID-19 and its associated pathophysiology in natural infection and vaccinated individuals. Therefore, it is really important to look at the specificity of the currently developed vaccine candidates for its effectiveness and protection. Majority of the currently developed vaccines are based on the spike glycoprotein; however, we should also focus on the importance of other structural and non-structural proteins for the development of effective COVID-19 vaccine. Emergence of SARS-CoV-2 variants imposes imperative concerns for COVID-19 vaccination due to their vaccine breakthrough cases. Therefore, the ideal vaccine may focus on the protection against various predominant SARS-CoV-2 variants and be able to induce effective memory T cell generation for long-term protection.

## Author Contributions

SS and SK conceived the idea. SK, VM, and SS collected the data, devised the initial draft, and reviewed the final draft. SS, SK, VM, and AT finalized the draft for submission. All authors contributed to the article and approved the submitted version.

## Conflict of Interest

The authors declare that the research was conducted in the absence of any commercial or financial relationships that could be construed as a potential conflict of interest.

## Publisher’s Note

All claims expressed in this article are solely those of the authors and do not necessarily represent those of their affiliated organizations, or those of the publisher, the editors and the reviewers. Any product that may be evaluated in this article, or claim that may be made by its manufacturer, is not guaranteed or endorsed by the publisher.
